# Overground walking speed changes when subjected to body weight support conditions for nonimpaired and post stroke individuals

**DOI:** 10.1186/1743-0003-7-6

**Published:** 2010-02-11

**Authors:** Jamie K Burgess, Gwendolyn C Weibel, David A Brown

**Affiliations:** 1 Department Of Physical Therapy and Human Movement Sciences, Northwestern University, Chicago, IL, USA; 2 Sensory Motor Performance Program, The Rehabilitation Institute of Chicago, Chicago, IL, USA

## Abstract

**Background:**

Previous research has shown that body weight support (BWS) has the potential to improve gait speed for individuals post-stroke. However, body weight support also reduces the optimal walking speed at which energy use is minimized over the gait cycle indicating that BWS should reduce walking speed capability.

**Methods:**

Nonimpaired subjects and subjects post-stroke walked at a self-selected speed over a 15 m walkway. Body weight support (BWS) was provided to subjects at 0%, 10%, 20%, 30%, and 40% of the subject's weight while they walked overground using a robotic body weight support system. Gait speed, cadence, and average step length were calculated for each subject using recorded data on their time to walk 10 m and the number of steps taken.

**Results:**

When subjected to greater levels of BWS, self-selected walking speed decreased for the nonimpaired subjects. However, subjects post-stroke showed an average increase of 17% in self-selected walking speed when subjected to some level of BWS compared to the 0% BWS condition. Most subjects showed this increase at the 10% BWS level. Gait speed increases corresponded to an increase in step length, but not cadence.

**Conclusions:**

The BWS training environment results in decreased self-selected walking speed in nonimpaired individuals, however self-selected overground walking speed is facilitated when provided with a small percentage of body weight support for people post-stroke.

## Background

Locomotor disability remains a major obstacle to community function in persons with chronic post-stroke hemiplegia. This disability is best characterized by a reduced gait speed that is observed in the majority of persons with post-stroke hemiplegia and has been shown to be correlated with other parameters such as balance, use of walking aids, number of falls, and ability to perform activities of daily living [1]. Rehabilitation programs often tackle the challenge of gait training post stroke with one or more interventions that include overground gait training, body weight support treadmill training (BWSTT), and/or strength training [2]. For example, body weight support has been utilized over a treadmill with the goal to unload a percentage of body mass and provide external support so that weight shifting, balance and stepping can be guided by the clinician at regulated speeds [3]. A primary motivation of this particular therapeutic method is to improve gait speed for people post stroke [4]. However, there is limited literature that explores how overground walking speed is altered while subjected to the body weight support environment for people post stroke during walking.

For people with an intact nervous system, supporting a percentage of body weight during walking would theoretically slow gait speed due to the minimization of energy expenditure across the gait cycle optimally occurring at a lower speed [5-7]. The determination of this optimal comfortable walking speed depends on several factors such as leg length, limb stiffness, and body load [8]. Subsequently, energy expenditure occurs optimally at a reduced speed as a result of reduced body load [8].

Despite the biomechanical evidence that a reduced speed for a nonimpaired person might occur while subjected to body weight support during walking, there are possible reasons why walking speed for someone with post-stroke hemiplegic gait might be facilitated during the BWS condition. For instance, since an individual with hemiplegia due to stroke injury walks with a slow speed, the reduction in net body weight could allow for a greater ability to propel the body forward when there is a weakness in one of the legs since there is a direct relationship between preferred walking speed and paretic leg propulsive impulse [9,10]. Additionally, increases in walking speed often correspond to a larger proportion of the gait cycle spent in single stance [11]. Body weight support would relieve loading on legs during the single support phase allowing an individual with stroke to remain in that phase longer and lessen the amount of time in double stance [11]. Finally, one of the primary motivations of body weight support treadmill training is the assumption that it facilitates the rhythmic spinal neuron pools for people post stroke [12]. Since the motor output of these central pattern generators is shaped by afferent feedback [13], BWSTT is hypothesized to promote improved walking function through the repetitive input of task-specific sensory feedback [14].

In summary, biomechanical evidence suggests that reduced speed might occur when subjected to body weight support during walking for a nonimpaired person, but an increased walking speed might occur for someone with post-stroke hemiplegic gait. We tested these predictions by exploring changes in self selected walking speed when subjected to body weight support for these two populations. We hypothesized that self selected walking speed for nonimpaired subjects would show a decreasing trend with increasing levels body weight support. In contrast, subjects post stroke would show an increase in self selected walking speed at some level of body weight support when compared with walking overground with no body weight support.

## Methods

### Subjects

Eleven neurologically nonimpaired subjects aged 40-72 (mean = 50, SD = 9) and twelve subjects aged 27-68 (mean = 52, SD = 12) presenting with chronic stroke consented to participate in this study. Relevant criteria for recruitment of nonimpaired subjects were as follows: over 40 years in age, no history of cardiac disease that would prevent them from participating in mild exercise, and able to walk 10 m unassisted. Relevant clinical measures are presented in Table 1 for the subjects post stroke. For this group, inclusion criteria consisted of unilateral stroke greater than 12 months past onset resulting in hemiplegia, medically approved for physical therapy, and were partially ambulatory such that a 15 m walk could be completed without the use of an assistive device other than an ankle-foot orthosis. The exclusion criteria were limited to the following: severe cardiac disease, a history of premorbid gait disorder of any cause, and an inability to follow simple commands. To characterize the group of subjects post stroke, subjects completed the Berg Balance Test and Lower Limb Fugl Meyer exam less than a week prior to completing the experimental protocol. This study was performed at the Rehabilitation Institute of Chicago and informed consent was obtained according to the policies of Northwestern University Institutional Review Board. Nonimpaired subjects were associated with the Rehabilitation Institute of Chicago and subjects post stroke were recruited through the Rehabilitation Institute of Chicago's Stroke Research Registry. Recruitment and clinical testing was completed by a research physical therapist.

**Table 1:** Clinical Features of Subjects Post Stroke

Subject	Age (Years)	Side of Paresis	Months Post Stroke	Berg Balance Score	Fugl Meyer Score	6 min walk speed (m/s)
1	65	L	247	44	13	0.58
2	58	L	134	55	27	1.25
3	46	L	168	40	8	0.5
4	68	R	26	50	23	0.98
5	59	R	51	44	14	0.81
6	37	L	27	41	12	0.34
7	27	L	42	56	26	1.06
8	45	L	27	45	9	0.53
9	54	L	240	45	15	0.62
10	60	R	64	53	13	0.35
11	53	R	74	45	14	0.35
12	55	R	264	56	19	1.06

Mean:	52	-	114	48	16	0.7
SD:	12	-	93	6	6	0.32

### KineAssist Gait and Balance Training System

**Figure 1:**
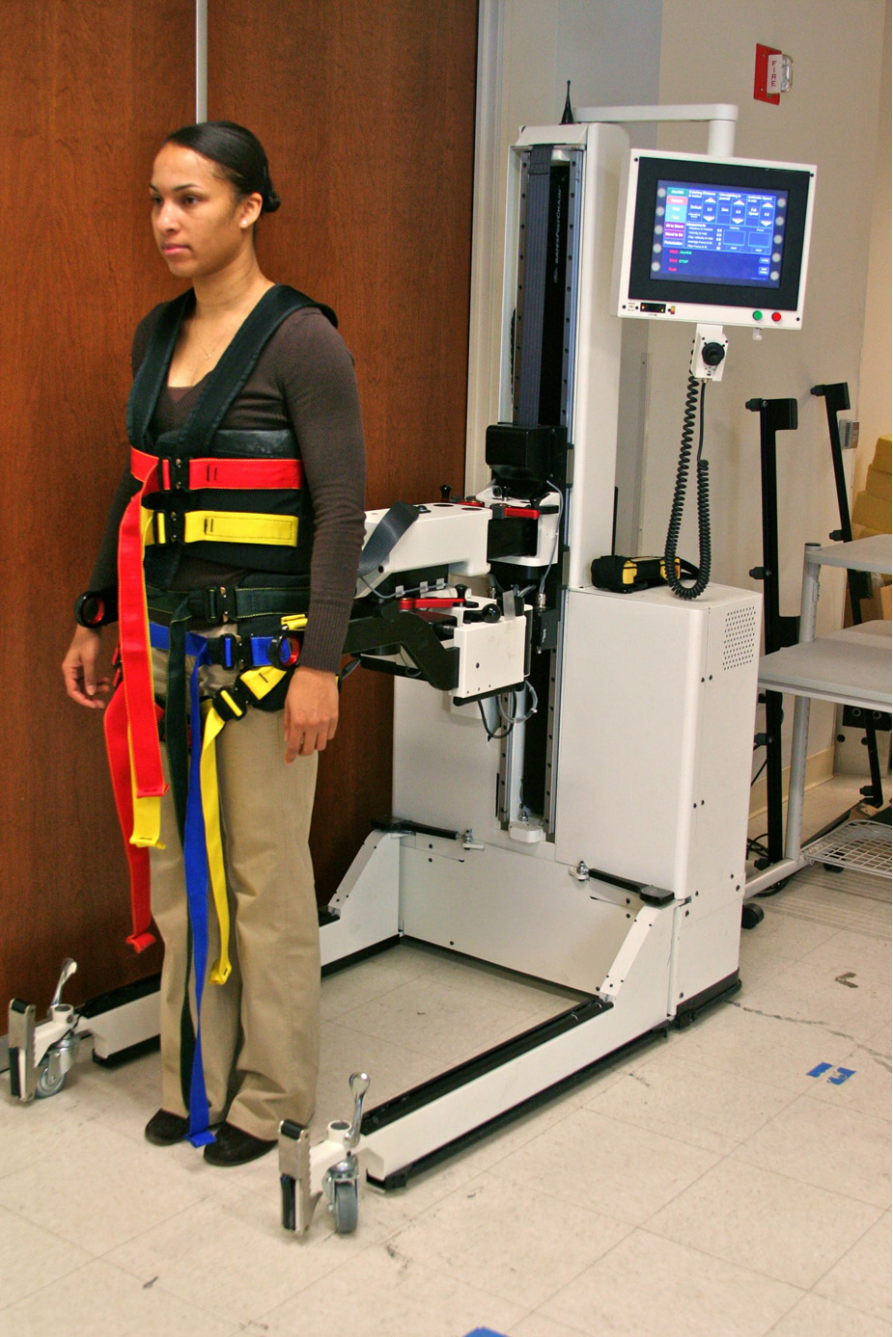
**The KineAssist in use**.

The KineAssist Gait and Balance Training System™ consists of a custom designed torso and pelvis harness attached to a mobile robotic base (Fig 1). The KineAssist utilizes a servomechanism to drive the robot according to the forces detected from the subject by the load cells located in the pelvic harness. This device has been described extensively elsewhere [15]. Patton et al., characterized the effect of this device on walking speed and found that it slows walking speed while still allowing the user to maintain normal walking kinematics [16]. This manner of admittance control slightly slows the user in order to promote safety and stability [16]. To accomplish overground walking with body weight support, the KineAssist offers closed loop body weight support continuously throughout the gait cycle, while the individual walks over ground. The vertical column provides this body weight support continuously while still allowing vertical movements of the pelvis.

### Protocol of Experiment

A 15 m track was set up for this experiment using tape to demark the straight-line path subjects were to follow. Only the performance during the middle 10 m were used in data collection; the first and last 2.5 m were used as buffer zones to avoid reporting the gait changes associated with starting and stopping gait. The 10 m distance was selected as the evaluation distance due to its common use in assessing comfortable walking speed in a clinical setting. It is also short enough to avoid the negative effects of fatigue for the subjects post stroke. Subjects were encouraged to walk as they normally would at a comfortable pace for every trial. For both subject groups, the first experimental trial consisted of walking 15 m unaided by the KineAssist while time to walk 10 m was recorded with a stopwatch and number of steps was manually counted within the 10 m length. For the purposes of this study, steps taken while the foot was planted on the start and/or finish line were included. A pseudo-random level of body weight support was presented to the subject via the KineAssist ranging from 0% BWS to 40% BWS, at 10% intervals, during subsequent trials for the nonimpaired subjects. BWS levels were randomized using Matlab™ for each subject. Subjects post stroke were presented with sequentially increasing levels of BWS over the same range and intervals of BWS. This was done because subjects post stroke showed discomfort when presented with intervals of BWS larger than 10% presented between trials. Additionally, one subject post stroke was unable to complete the task at the 40% BWS level due to an inability to maintain a normal walking pattern for this subject. The final trial for both subject groups was a repetition of the first trial wherein subjects walked 15 m without the use of the KineAssist.

### Data Analysis

The speed for each trial was computed using the distance of 10 m divided by the recorded time to complete that distance. Average step length was calculated by dividing the 10 m distance by the number of steps recorded. Cadence was calculated by dividing the number of steps taken during the 10 m by the time needed to complete the 10 m.

We normalized all of the speed, cadence, and average step length data that were collected during the trials completed with the use of the KineAssist. This normalization had the primary effect of allowing evaluation of intrasubject changes in self selected walking speed with BWS and removed the large variability seen in self selected walking speeds. Each variable measured at the 10%-40% BWS level was divided by the same variable measured at the 0% BWS level and was expressed as a percentage change from the 0% BWS level. From this normalization protocol, we directly determined if a subject had increased or decreased his or her self selected walking speed with BWS from the 0% BWS level. For example, if the normalized percentage change in speed was positive, then an increase in self selected walking speed at the corresponding level of BWS would have occurred.

Statistical analyses were completed on the normalized data. One sample t-tests were completed to test the hypothesis that there were significant changes from the 0% BWS condition for each level of body weight support for velocity, cadence and average step lengths for each group. Significance was evaluated at P < 0.05. Data values are presented as the mean ± standard deviation. Plots are shown with confidence intervals.

We determined the maximum percent increase in velocity and maximum percent decrease in velocity for both subject groups by detecting the maximum values at any of the body weight support conditions (10%-40% BWS). We averaged the step length and cadence values associated with the maximum velocity values in order to determine which of these two factors might explain the increased walking velocity values. Additionally, we generated a histogram to examine the frequency with which the maximum velocity for each subject occurred at each body weight support level for each subject group.

## Results

### Nonimpaired subjects

**Figure 2:**
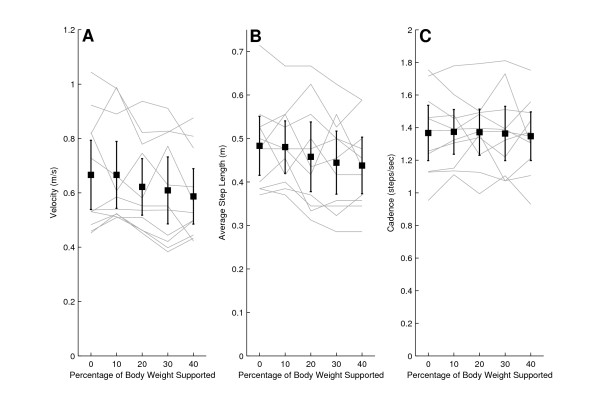
**Velocity (A), average step length (B), and cadence (C) for non-impaired subjects walking at different levels of BWS**. The dark solid line is the mean and 95% confidence intervals for all nonimpaired subjects. The lighter solid lines represent individual subject data.

When nonimpaired subjects walked 10 m without the use of the KineAsssist, their mean self selected walking speed was 0.5 m/s greater than walking in the KineAssist without any BWS (1.2 ± 0.2 m/s mean walking speed overground, 0.7 ± 0.2 m/s mean walking speed overground using KineAssist with no BWS). The nonimpaired subjects' self selected overground walking speed when walking with the KineAssist showed a downward trend as BWS increased (Fig 2, A). Additionally, average step length showed a decreasing trend as level of BWS increased. Cadence remained steady across all trials (Fig 2, B and 2C).

**Figure 3:**
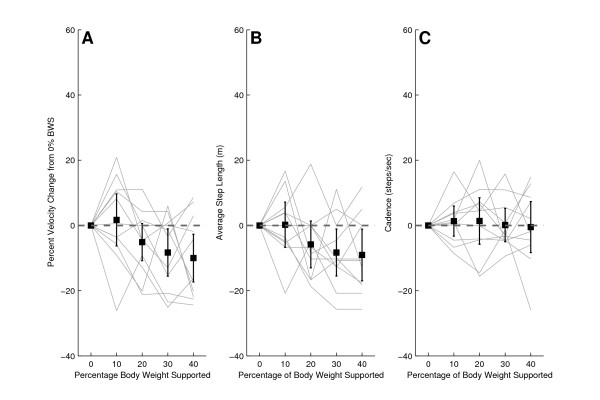
**Normalized velocity (A), average step length (B), and cadence (C) for non-impaired subjects walking at different levels of BWS**. The dark solid line is the mean and 95% confidence intervals for all nonimpaired subjects. The lighter solid lines represent individual subject data.

Each self selected walking speed, cadence, and step length measure recorded in the KineAssist was normalized to the respective measure obtained at the 0% BWS level in the KineAssist. The normalized data showed a decline in changes in self selected walking speed in the KineAssist from the 0% BWS level as BWS levels were increased (Fig 3, A). A linear regression with a least squares fit of individual subject data resulted in an average slope of -0.0038 ± 0.008%velocity change/%BWS (p < 0.05). Additional analysis of the y-intercept revealed that the estimated y-intercept was not significantly different from 0% (y = 0.04, p > 0.05). This value was expected since every value was normalized to 0% velocity change at 0% BWS. Additionally, the average step length with BWS also decreased with a slope of -0.03 ± 0.0003 m/%BWS (p < 0.05) and cadence varied little across different levels of BWS (Fig 3, B and 3C).

**Figure 4:**
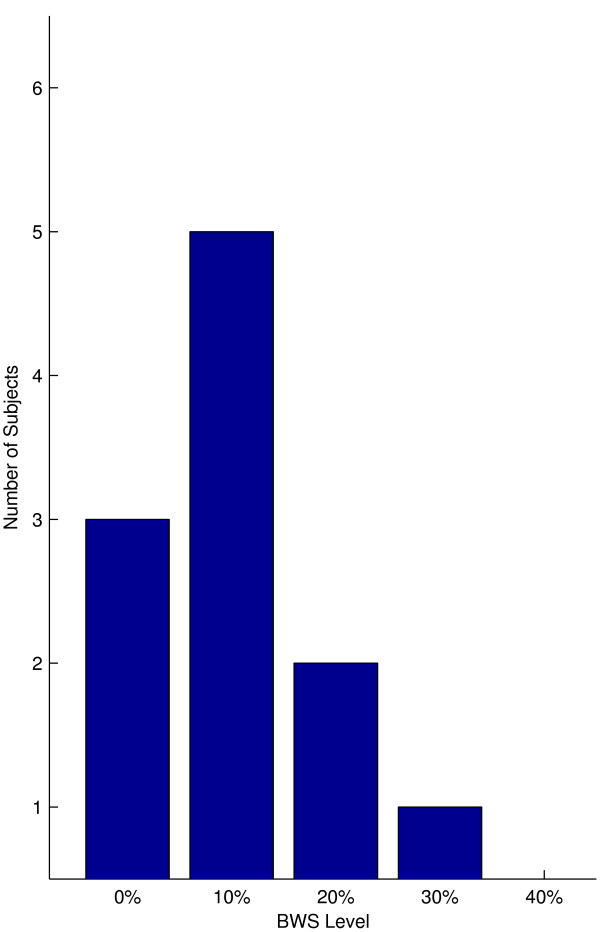
**Number of nonimpaired subjects that attained the maximum percentage change in velocity at each level of BWS**.

A histogram of how many subjects attained their maximum speed increase in the KineAssist at each level of BWS showed that there was a skew towards the 10% BWS level despite a lack of a significant increase in self selected walking speed in the KineAssist at that level (Mean velocity percentage change at 10% BWS = 1.02 ± 0.13%, p > 0.05 Fig 4).

### Subjects post-stroke

The average self selected walking speed without the use of the KineAssist for subjects post stroke was 0.8 ± 0.3 m/s. When using the KineAssist with no BWS, the average self-selected walking speed was 0.4 ± 0.1 m/s. Average step length also shortened from 0.5 ± 0.08 m without the use of the KineAssist to 0.3 ± 0.08 m when using the KineAssist with 0% BWS.

**Figure 5:**
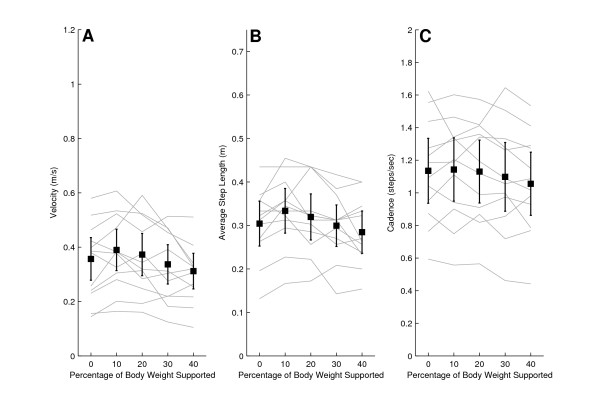
**Velocity (A), average step length (B), and cadence (C) for post-stroke subjects walking at different levels of BWS**. The dark solid line is the mean and 95% confidence intervals for all post-stroke subjects. The lighter solid lines represent individual subject data.

The self selected walking speed data collected in the KineAssist from subjects post-stroke showed high variability among subjects as expected (Fig 5, A). This high variability was also apparent with the average step length, and cadence data (Fig 5, A, B, and 5C, respectively).

**Figure 6:**
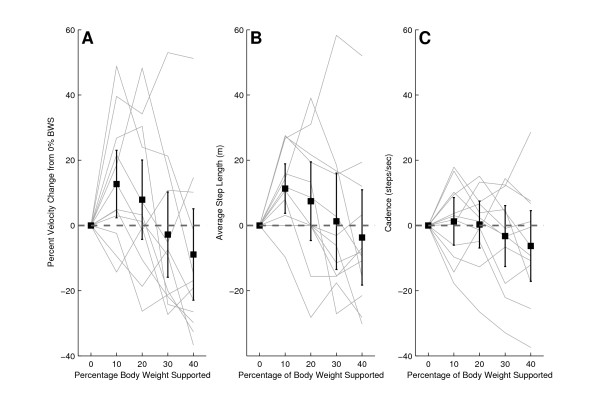
**Normalized velocity (A), average step length (B), and cadence (C) for post-stroke subjects walking at different levels of BWS**. The dark solid line is the mean and 95% confidence intervals for all post-stroke subjects. The lighter solid lines represent individual subject data.

Upon normalization, the mean across subjects of the self selected walking speed in the KineAssist showed a 13% increase at the 10% BWS level over the self selected walking speed in the KineAssist at the 0% BWS level (Fig. 6, A; 1.13 ± 0.18%, p < 0.05). Higher levels of BWS did not elicit any significant speed increases from the 0% BWS level speeds for subjects post-stroke. Normalized average step length in the KineAssist showed an 11% increase from the 0% BWS level at the 10% BWS level (Fig 6, B and 6C).

Examination of the slopes of the individual self selected walking speeds in the KineAssist over the 10%-40% BWS levels revealed that the mean slope was significantly different than 0 (mean slope = -0.08 ± 0.008% velocity change/% BWS, p < 0.05). Additionally, the y-intercept was also significantly different than 0% (y-intercept = 1.211 ± 0.02, p < 0.05).

**Figure 7:**
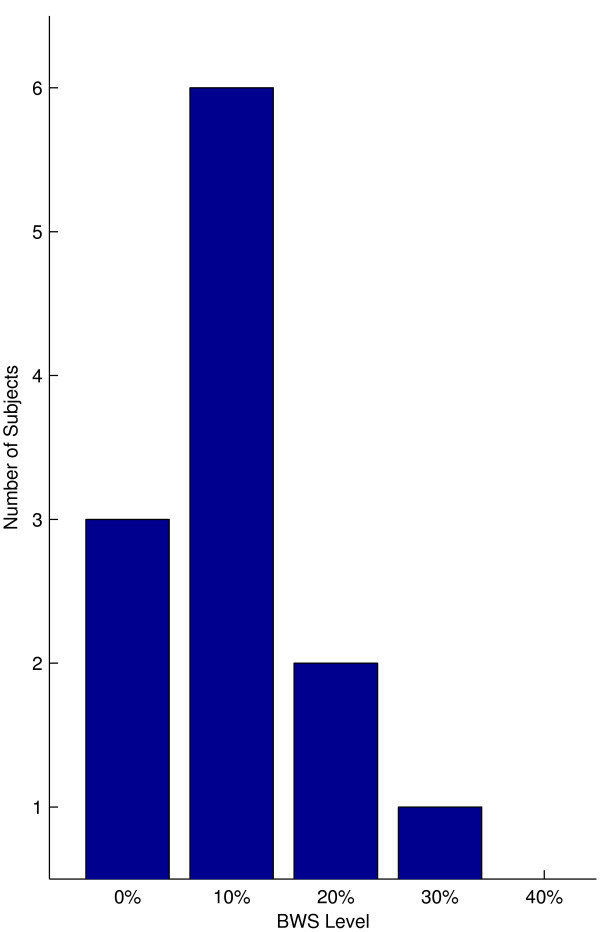
**Number of post-stroke subjects that attained the maximum percentage change in velocity at each level of BWS**.

Despite the group mean of self selected walking speed changes in the KineAssist showing a statistically significant increase at the 10% level of BWS, individual subjects showed maximum self selected walking speed increases in the KineAssist at a variety of BWS levels. At least half of the subjects had their maximum KineAssist walking speed at a BWS level other than 10% (Fig 7) and three subjects did not show an increase in speed at any level of BWS over the 0% BWS level.

**Figure 8:**
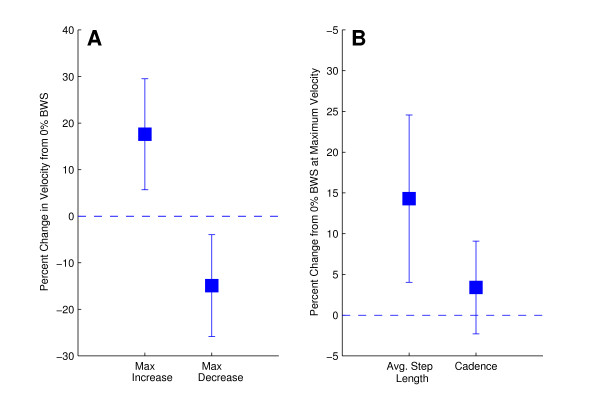
**Plot A indicates the maximum percent increase and maximum percent decrease in velocity that post-stroke subjects attained regardless of level of support**. Plot B examines the percent change in average step length and cadence recorded at a subject's maximum speed for the post-stroke subjects.

We grouped all BWS conditions together to calculate the mean maximum percent increase and maximum percent decrease in self selected walking speed in the KineAssist over all levels of BWS. There was a significant increase in self selected walking speed in the KineAssist at any level of BWS (Fig 8, A, mean maximum percent increase in speed = 17.5 ± 21.0%, p < 0.05). Also, an examination of the maximum percent decrease in self selected walking speed in the KineAssist showed that subjects post-stoke experienced a significant decrease in walking speed at any level of BWS (Fig 8A, mean maximum percent decrease = 14.9 ± 19.3%; p < 0.05 and p < 0.05 respectively).

Subjects significantly increased their average step length in the KineAssist at the maximum speed attained for each subject when compared to each subjects' average step length at the 0% BWS level (Fig 8B, 14.3 ± 18.1% p < 0.05). The changes in cadence were not significantly different than zero percent change for any level of BWS (Fig 8B, p > 0.05).

## Discussion

The systematic decline in self selected walking speed in the KineAssist with increasing levels of BWS as seen in neurologically nonimpaired subjects was expected due to model predictions from a previous study that modelled the interaction between gravity and self-selected walking speed [17]. During the gait cycle, there is a continuous transformation of potential and kinetic energy. The efficiency of this transformation depends on the mass of the individual, the effect of gravity acting on that mass, and the speed that the mass is travelling [17]. When there is a reduction in the effective weight of an individual, the speed that the individual is walking ideally must be reduced in order to minimize the level of energy expenditure [18]. Since we obtained a reasonably linear relationship (R^2 ^= .96) between self-selected comfortable walking speed and level of BWS, the expected decrease is supported.

The self-selected walking speeds in the KineAssist of subjects with post-stroke hemiparesis showed variation from the prediction of the nonimpaired model in that their average percent velocity change in response to increasing levels of BWS over 0% BWS in the KineAssist, did not decrease but instead showed an increase at 10% BWS. There must be an alternate mechanism or mechanisms that people post-stroke utilize to determine self selected walking speed.

The data collected in this study was spatio-temporal in nature and, therefore, we acknowledge that we cannot suggest specific mechanistic causes that underlie these observations. However we propose three possible mechanisms that might account for the increase in self-selected walking speed in the KineAssist in post-stroke individuals when provided body weight support in support of future studies in regards to how BWS and sensory feedback regarding loading might alter and improve locomotion post stroke.

The first of these potential mechanisms is that BWS can compensate for paretic leg muscle weakness leading to improved propulsion and decreased asymmetry in force production. Weakness is a common issue that arises post-stroke that is characterized by a reduction in force production from muscle [10]. When body load is supported, weak muscles can better match the physical demands of locomotion potentially leading to a more energy efficient gait pattern [19]. Weight acceptance is also improved similarly for both legs with BWS [19]. Additionally, improved weight acceptance associated with BWSTT might reduce extensor spasticity associated with loading [20].

Secondly, load related sensory feedback is critical for generating effective gait mechanics in a nonimpaired nervous system [21], by promoting ongoing extensor activity during stance and facilitating phase transitions from stance to swing [13]. Stroke has the potential to degrade the ability of the spinal cord to appropriately utilize load related sensory signals leading to abnormal extensor activity during stance [22]. BWS may promote improved output through proper sensory facilitation of the locomotor neuron pools [3,23]. Less inappropriate drive from these sensory afferents onto spinal neural networks could improve the generation of appropriate control signals driving the muscles of the lower limbs during locomotion [24]. This improved processing could have a beneficial effect on accurate estimation of the location of the center of mass relative to the base of support across the gait cycle, thus facilitating gait mechanics.

Thirdly, reorganization of descending drive could play a role in affecting gait speed during weight-supported locomotion. Previous studies suggest that corticospinal descending pathways from the injured brain area become less effective at generating successful motor commands after stroke and that the nervous system relies more on indirect, brainstem mediated descending tracts that are often ill-suited for finer motor tasks [21,25,26]. In addition, there may be an increased reliance on the reticulospinal tract, which is more adept at controlling posture and gross movements of the trunk and proximal muscles [27]. This would result in a loss of independent joint control between the upper and lower limbs during dynamic weight bearing tasks such as walking [26]. Finally, work in animals has shown that there is a greater use of reticulo- and rubrospinal pathways after lesions of corticospinal tracts [28]. Indeed, studies that have provided weight support to the arm during reaching tasks for people with post stroke hemiplegia have found a reduction in inappropriate coupling of joint movements allowing for greater control of reaching movements [25].

People with post-stroke hemiparesis respond differently than nonimpaired individuals when walking overground with body weight support. Specifically, nonimpaired people show a gradual decline in self-selected walking speed in accordance with biomechanical models [17], but people post-stroke show an increase in self-selected walking speed at low levels of body weight support as shown in the data presented in this article. Further examination of biomechanical factors such as dynamic strength, ground reaction forces, and EMG activity could provide insight towards potential mechanisms by which this behavior occurs. However, of particular importance is this first step of observing the nonstandard behavior seen in the post-stroke subjects during self selected overground walking.

This study was constrained by several factors. The task of overground walking imposed a limit of how much body weight support we could supply to a subject that permitted them to be able to successfully walk forwards comfortably. This is in contrast to many previous body weight support studies that were performed over a treadmill where greater levels of BWS could be studied [4,29,30]. Despite this limitation, our results suggest that walking speeds would continue to be compromised at higher levels of BWS. Additionally, our observation of degradation of walking speed at higher levels of body weight support around 40% BWS supports results found in other studies that recommend using levels of BWS less than 30-45% [20,31].

For the subjects post-stroke, we were concerned about fatigue effects during the experiment so we only performed one trial at each BWS level. Additionally, we performed the tests at 10% BWS intervals. However, further examination of behaviour around the 10% and 20% BWS levels at smaller increments would be useful in further elucidating any possible trend. Additionally, the subjects post stroke received the BWS in increasing increments of BWS due to discomfort they experienced when transitioning between increments of BWS higher than 10%. We were concerned that there might be a learning effect between trials but since we did not see a constant increase in self selected walking speed in the KineAssist with increasing levels of BWS, we were sufficiently satisfied that any possible learning effect was not apparent in our data.

We also found that about half of the nonimpaired subjects had difficulty maintaining a normal gait pattern during higher levels of BWS. Several subjects would attempt a "loping gait" at 30% and 40% BWS levels. This gait is characterized by long upward jumps between steps similar to the gait maintained by astronauts walking on the moon [17]. This loping gait was detected prior to data collection and the trial would be restarted and instructions regarding maintaining a typical gait pattern would be emphasized. One nonimpaired subject was excluded because he was not able to maintain a walking gait but instead performed this jumping gait. The subjects with post-stroke hemiparesis were never observed performing a loping gait and were able to complete all trials without major deviations to the gait pattern seen at the 0% BWS level.

Finally, we did not explore kinetic variables since we were simply looking to explore overground self selected speed output based on the amount of BWS provided. We felt that self selected walking speed would reflect global locomotor fitness and if there was significant differences in behavior. Further experiments will be examining ground reaction forces and electromyographic variables to deeper explore how increases in speed for people post-stroke evolve when provided body weight support.

## Conclusions

This study found that subjects with post-stroke hemiparesis increased their overground self-selected walking speeds in the KineAssist by an average of 17% when walking with some level of body weight support compared with their self selected walking speed with 0% BWS. Conversely, when neurologically nonimpaired subjects performed the same task, their self selected walking speeds in the KineAssist decreased at all levels of BWS when compared to their walking speeds with 0% BWS. With post-stroke subjects, increased self selected walking speed in the KineAssist was associated with increased average step length by 14%, whereas cadence did not change significantly over any level of BWS. Although each individual subject post-stroke showed increased walking velocities at different BWS levels, the 10% BWS level was the only condition that showed a significant group average increase in speed over the 0% BWS level.

While we did not find a defining characteristic that indicates the subjects that would best benefit from this type of training or what level of body weight support would be most appropriate, we did find that most of the stroke subjects did walk faster with some level of body weight support. Further research is necessary in order to determine the possible load related mechanisms that influence gait speed for subjects post stroke in order to inform gait rehabilitation research.

## Competing interests

DB participated as a consultant with the startup company KineaDesign, LLC, the company that designed and build the KineAssist device. He is listed as an inventor who will potentially receive Royalty Payments.

## Authors' contributions

JB carried out data collection, analysis and drafted the manuscript. GW assisted in data collection, completed subject recruitment, and helped draft the manuscript. DB participated in the design of the study, statistical analysis, and drafting the manuscript. All authors read and approved the final manuscript.

## References

[B1] Potter JM Evans AL Gait Speed and Activities of Daily Living Function in Geriatric Patients Archives of Physical Medicine and Rehabilitation 1995 76 997 999 10.1016/S0003-9993(95)81036-6 7487453

[B2] Hesse S Werner C Bardeleben A Barbeau H Body Weight-Supported Treadmill Training After Stroke Current Artherosclerosis Reports 2001 3 287 294 10.1007/s11883-001-0021-z 11389793

[B3] Hassid E Rose D Commisarow J Guttry M Dobkin BH Improved Gait Symmetry in Hemiparetic Stroke Patients Induced During Body Weight-Supported Treadmill Stepping Neurorehabilitation and Neural Repair 1997 11 21 26 10.1177/154596839701100104

[B4] Visintin M Barbeau H Korner-Bitensky N Mayo N A new approach to retrain gait in stroke patients through body weight support and treadmill stimulation Stroke 1998 29 1122 9626282 10.1161/01.str.29.6.1122

[B5] Cavagna GA Saibene FP Margaria R External Work in Walking Journal of Applied Physiology 1963 18 1 9 14019429 10.1152/jappl.1963.18.1.1

[B6] Cavagna GA Thys H Zamboni A The Sources of External Work in Level Walking and Running The Journal of Physiology 1976 262 639 657 1011078 10.1113/jphysiol.1976.sp011613 PMC1307665

[B7] Waters RL Lunsford BR Perry J Byrd R Energy-speed Relationship of Walking: Standard Tables Journal of Orthopaedic Research 1988 6 215 222 10.1002/jor.1100060208 3343627

[B8] Cavagna G Willems P Heglund N The role of gravity in human walking: pendular energy exchange, external work and optimal speed The Journal of Physiology 2000 528 657 668 10.1111/j.1469-7793.2000.00657.x 11060138 PMC2270143

[B9] Bowden MG Balasubramanian CK Neptune RR Kautz SA Anterior-Posterior Ground Reaction Forces as a Measure of Paretic Leg Contribution in Hemiparetic Walking Stroke 2006 37 872 876 10.1161/01.STR.0000204063.75779.8d 16456121

[B10] Olney S Richards C Hemiparetic gait following stroke. Part I: Characteristics Gait & Posture 1996 4 136 148

[B11] Lamontagne A Faster Is Better: Implications for Speed-Intensive Gait Training After Stroke Stroke 2004 35 2543 2548 10.1161/01.STR.0000144685.88760.d7 15472095

[B12] Hesse S Konrad M Uhlenbrock D Treadmill walking with partial body weight support versus floor walking in hemiparetic subjects Archives of Physical Medicine and Rehabilitation 1999 80 421 427 10.1016/S0003-9993(99)90279-4 10206604

[B13] Pearson KG Proprioceptive regulation of locomotion Neurobiology 1995 1 6 8805415 10.1016/0959-4388(95)80107-3

[B14] Sullivan K Knowlton B Dobkin B Step training with body weight support: effect of treadmill speed and practice paradigms on poststroke locomotor recovery Archives of Physical Medicine and Rehabilitation 2002 83 683 691 10.1053/apmr.2002.32488 11994808

[B15] Peshkin M Brown DA Santos-Munne JJ Makhlin A Lewis E Colgate JE Patton J Schwandt D KineAssist: A robotic overground gait and balance training device Proceedings of the 2005 IEEE ICORR 2000 1 6 10.1310/tsr1502-131 18430678

[B16] Patton J Brown D Lewis E Crombie G Santos-Munne JJ Makhlin A Colgate JE Peshkin M Motility evaluation of a novel overground function mobility tool for post stroke rehabilitation Proceedings of the 2007 IEEE 10th International Conference on Rehabilitation Robotics 2007 1049 1054 full_text

[B17] Cavagna GA Willems PA Heglund NC The Role of Gravity in Human Walking: Pendular Energy Exchange, External Work and Optimal Speed The Journal of Physiology 2000 528 657 668 10.1111/j.1469-7793.2000.00657.x 11060138 PMC2270143

[B18] Cavagna GA Thys H Zamboni A The sources of external work in level walking and running Journal of Physiology 1976 1 19 10.1113/jphysiol.1976.sp011613 PMC1307665 1011078

[B19] Roopchand S Fung J Barbeau H Locomotor training and the effects of unloading on overground locomotion following stroke 2005 Hauppauge: Nova Science Publishers

[B20] Hesse S Helm B Krajnik J Gregoric M Mauritz KH Treadmill Training with Partial Body Weight Support: Influence of Body Weight Release on the Gait of Hemiparetic Patients Neurorehabilitation and Neural Repair 1997 11 15 20 10.1177/154596839701100103

[B21] Dietz V Proprioception and locomotor disorders Nat Rev Neurosci 2002 3 781 790 10.1038/nrn939 12360322

[B22] Reinkensmeyer D How to retrain movement after neurologic injury: a computational rationale for incorporating robot (or therapist) assistance Proceedings of the 2003 IEEE Engineering in Medicine and Biology Society Meeting 2003 2 1479 1482

[B23] Dietz V Duysens J Significance of load receptor input during locomotion: a review Gait & Posture 2000 11 102 110 10.1016/s0966-6362(99)00052-1 10899663

[B24] Miyai I Suzuki M Hatakenaka M Kubota K Effect of body weight support on cortical activation during gait in patients with stroke Exp Brain Res 2006 169 85 91 10.1007/s00221-005-0123-x 16237521

[B25] Beer RF Ellis MD Holubar BG Dewald JPA Impact of gravity loading on post-stroke reaching and its relationship to weakness Muscle Nerve 2007 36 242 250 10.1002/mus.20817 17486581 PMC2866301

[B26] Kline TL Schmit BD Kamper DG Exaggerated interlimb neural coupling following stroke Brain 2006 130 159 169 10.1093/brain/awl278 17018550

[B27] Schepens B Drew T Independent and convergent signals from the pontomedullary reticular formation contribute to the control of posture and movement during reaching in the cat Journal of Neurophysiology 2004 92 2217 10.1152/jn.01189.2003 15175364

[B28] Riddle CN Edgley SA Baker SN Direct and indirect connections with upper limb motoneurons from the primate reticulospinal trac The journal of Neuroscience 2009 29 4993 4999 10.1523/JNEUROSCI.3720-08.2009 19369568 PMC2690979

[B29] Hesse S Bertelt C Jahnke MT Schaffrin A Baake P Malezic M Mauritz KH Treadmill Training with Partial Body Weight Support Compared with Physiotherapy in Nonambulatory Hemiparetic Patients Stroke 1995 26 976 981 7762049 10.1161/01.str.26.6.976

[B30] Teixeira I Lim PA Qureshy H Henson H Monga T Protas EJ A Comparision of Regular Rehabilitation and Regular Rehabilitation with Supported Treadmill Ambulation Training for Acute Stroke Patients Journal of Rehabilitation Research and Development 2001 38 245 255 11392657

[B31] Dobkin BH An Overview of Treadmill Locomotor Training with Partial Body Weight Support: A Neurophysiologically Sound Approach Whose Time Has Come for Randomized Clinical Trials Neurorehabilitation and Neural Repair 1999 13 157 165 10.1177/154596839901300301

